# Targeted Inhibition of mTOR Signaling Improves Sensitivity of Esophageal Squamous Cell Carcinoma Cells to Cisplatin

**DOI:** 10.1155/2014/845763

**Published:** 2014-04-10

**Authors:** Guiqin Hou, Shuai Yang, Yuanyuan Zhou, Cong Wang, Wen Zhao, Zhaoming Lu

**Affiliations:** ^1^School of Pharmaceutical Sciences, Zhengzhou University, 100 Kexue Avenue, Zhengzhou, Henan 450001, China; ^2^New Drug Research and Development Centre of Zhengzhou University, Zhengzhou, Henan 450001, China

## Abstract

mTOR is an evolutionarily conserved serine-threonine kinase with a central role in cell growth, invasion, and metastasis of tumors, and is activated in many cancers. The aims of this study were to investigate the expression of mTOR in ESCC tissues and its relationship with progression of ESCC and measure the changes of sensitivity of ESCC cells to cisplatin after cells were treated with mTOR siRNA by WST-8 assays, TUNEL, RT-PCR, and western blots in vitro and in vivo. The results showed that the expression of mTOR was higher in ESCC specimens than that in normal esophageal tissues and its expression was closely correlated with the TNM stage of ESCC. mTOR siRNA significantly increased the sensitivity of the EC9706 cells to cisplatin at proliferation in vitro and in vivo. The growth of ESCC xenografts was significantly inhibited by mTOR siRNA or cisplatin, and the cell number of apoptosis was obviously increased after xenografts were treated with mTOR siRNA or cisplatin alone, especially when mTOR siRNA combined with cisplatin. The present study demonstrates that the expression of mTOR has important clinical significance and inhibition of mTOR pathway by mTOR siRNA can improve the sensitivity of ESCC cells to cisplatin.

## 1. Introduction

Esophageal squamous cell carcinoma (ESCC) is one of the most frequently diagnosed cancers in developing countries, especially in Northern China [1], and patients with ESCC have a poor prognosis with a dramatic decreased 5-year survival rate [2, 3]. It is thus imperative to find new therapeutic targets underlying initiation and progression of ESCC to improve therapy for ESCC.

Mammalian target of rapamycin (mTOR) is a member of the phosphoinositide 3-kinase-related kinase (PIKK) family with homologs in all mammalians and its activity has been linked with cell growth, proliferation, survival, protein translation, and other cellular metabolic processes [4–6]. Activation of mTOR occurs via a multistep process that includes upstream phosphoinositide 3-kinase (PI3K) and Akt activation [7, 8]. Activation of mTOR regulates a number of its downstream effectors important in cellular growth, such as p70S6 kinase (S6K) and elongation initiation factor 4E (eIF4E) binding protein-1 (4EBP1), resulting in enhanced translation of subset of genes that are required for protein synthesis and cell growth [9–11]. Accumulating evidences have demonstrated that mTOR has a central role not only for cell growth but also for invasion and metastasis of cancers [7]. Rapamycin is the special inhibitor of mTOR; more and more reports have shown that rapamycin and its anologs temsirolimus (CCI-779) and everolimus (RAD001) exert antiproliferative effects through the inhibition of mTOR by binding to FKBP12 [12, 13]. The inhibition of mTOR decreases phosphorylation and activation of p70S6K and 4EBP1, which results in the inhibition of translation of critical mRNA involved in tumorigenesis [4, 6]. Activation of mTOR pathway occurs in many cancers and has recently been shown to be correlated with more aggressive disease behavior [14, 15]. It has been assumed that this may be because mTOR at the crossroad of a network of molecular pathways regulates the synthesis of proteins required for growth of cancer cells [16]. At present, rapamycin and its analogs have been used in numerous clinical trials for solid tumor, such as prostate, breast, and pancreatic cancers, and they display encouraging antitumor activity with minimal toxicity and no immunosuppression over a broad of dose level [17].

In this study, the expression level of mTOR was examined by immunohistochemistry in human ESCC specimens, and the effects of mTOR siRNA and cisplatin alone or combined on cell proliferation, tumor growth, and cell apoptosis were, respectively, investigated in EC9706 cells and xenografts.

## 2. Materials and Methods

### 2.1. Patients and Tissue Samples

35 ESCC tissue samples (16 men and 19 women with the mean age of 61.3 ± 9.1 years) from Chinese patients were collected from Cancer Hospital of Anyang City, China. No patients had undergone chemotherapy or radiotherapy prior to surgery. Among them, histopathology classification was 9 (I), 14 (II), and 12 tissues (III), and the infiltration appeared in mucosa, muscle layer, and fiber membrane of 7, 15, and 13 tissues, respectively. Furthermore, lymph node metastasis existed in 16 of 35 patients, and TNM phase was I-II of 13 and III-IV of 22, respectively. After the tissues were fixed in 10% formalin and embedded in paraffin wax and 4 *μ*m thick sections were cut, the expressions of mTOR in them were measured immunohistochemically and the relationship between expression levels of mTOR protein and differentiation degree, depth of infiltration, lymph node metastasis, and TNM stage was analyzed, respectively.

### 2.2. Cell Culture and Animal Treatment

EC9706 cells purchased from the Chinese Academy of Medical Sciences, Beijing, China, were cultured in RPMI/1640 medium (Gibco-BRI, USA) supplemented with 10% fetal bovine serum (FBS) (Hyclone Laboratories, USA), 100 U/mL penicillin, and 100 *μ*g/mL streptomycin at 37°C in the presence of 5% CO_2_ as described previously [18].

All animal studies were carried out in compliance with the Guide for the Care and Use of Laboratory Animals of Henan Province, China. Male athymic BALB/c nude mice (Vital River Animal Ltd., Beijing, China) at 4-5 weeks of age were used in the study. Five mice per cage were housed in wire-top cages with sawdust bedding in an isolated, clean, air-conditioned room at a temperature of 25-26°C and a relative humidity of ~50%, lit 12 hours/day.

### 2.3. Immunohistochemical Analysis

The tissue sections were deparaffinized in xylene, hydrated through graded ethanols and distilled water, and washed thoroughly with PBS. For antigen retrieval, the slides were put in a container having 10 mmol/L citrate buffer (pH 6), and then the container was placed in boiled water for 20 minutes. After the slides were put at room temperature (RT) for 30 minutes, they were rinsed thrice with PBS. Subsequently the slides were incubated in 3% hydrogen peroxide in PBS for 30 minutes to quench the endogenous peroxidase. The sections were washed in PBS and then incubated in the blocking solution (10% rabbit serum in PBS) for 30 minutes in a chamber with saturated humidity at RT. Excess solution was discarded and the sections were incubated with primary antibody rabbit polyclonal anti-mTOR of 1 : 200 (Santa Cruz Biotechnology, USA) and PBS as negative control, respectively, at 4°C overnight. The slides having been washed with PBS were subsequently incubated with the biotinylated secondary antibody of 1 : 8,000 (Santa Cruz Biotechnology, USA) for 30 minutes, followed by being incubated with the HRP-linked streptavidin biotin complex in a box with saturated humidity for 10 minutes at RT. Finally, the slides were washed and developed with 3,3-diaminobenzidine (DAB) solution for ~3 minutes, and then the sections were counterstained with hematoxylin, dehydrated and cleared in xylene, and mounted. Immunohistochemical evaluation was performed by a pathologist without knowledge of the clinical and pathologic characteristics of these patients. The tumor cells were scored further according to the intensity (I), distribution (D), and pattern (P) reported by Dong et al. [19]: I score: 0, negative; 1, weak; 2, moderate; and 3, strong; D score (%): 0, negative; 1, 10–50%; 2, 51–90%; and 3, >90%; P score: 0, no staining; 1, sporadic positive staining; 2, focal positive staining; and 3, diffuse positive staining. The total scores of each tissue = I × D × P; the 0 score was negative, and ≥1 score was positive.

### 2.4. Cell Proliferation Assay

Cell proliferation was determined using WST-8 dye (Beyotime Inst Biotech, China) according to the manufacturer's instructions. Briefly, EC9706 cells transfected with mTOR siRNA (sc-35409, Santa Cruz) for 24 hours were harvested and seeded in a 96-well flat-bottomed plate (5 × 10^3^cells/well) and cultured at 37°C for 24 hours. Subsequently, cells were treated with cisplatin at increasing concentrations in the presence of 10% FBS for 24 hours. After 10 *μ*L WST-8 was added to each well, cells were incubated at 37°C for 2 hours and the absorbance was finally determined at 450 nm using a microplate reader (Bio-Rad Laboratories, USA). Each sample was assayed in triplicate for each group.

### 2.5. Tumor Xenografts in Athymic Nude Mice

EC9706 cells were treated with mTOR siRNA as indicated in our reported data [20]. Twenty athymic mice were divided into two groups of 10 mice each, in which one group was subcutaneously inoculated with EC9706 cells transfected with mTOR siRNA for 24 hours and another without mTOR siRNA. Briefly, cells grown at logarithm phase were harvested, washed, and resuspended in PBS at 2 × 10^7^cells/mL. A cell resuspension of 200 *μ*L (4 × 10^6^ cells) was inoculated s.c. into the right flank of athymic mice each. For tumor growth analysis, the tumor size was measured every day with a sliding caliper, and the tumor volume was defined as (longest diameter) × (shortest diameter)^2^/2 [21]. The cisplatin solution was prepared as described previously [18]. Further, tumor-bearing animals of the two groups were randomly subdivided into 2 groups of 5 animals each [22, 23], respectively. The treatment schedule was as follows. The groups without or with mTOR siRNA were injection i.p. with cisplatin (1 mg/kg) and PBS as controls, respectively, every day for 2 weeks. Inhibition rate = [(tumor volume of control group – tumor volume of experimental group)/tumor volume of control group] × 100%.

After being treated for two weeks, tumor-bearing mice were sacrificed and the tumors were removed, weighed, and then cut into 3 pieces, one of which immediately was fixed in 4% buffered paraformaldehyde overnight for TUNEL assay and two of which were put into liquid nitrogen solution for protein or RNA analysis.

### 2.6. Western Blots

Small pieces of fresh xenografts were immediately homogenized in protein lysis buffer and centrifugated at 12,000 rpm for 20 minutes, and then the supernatant was harvested as the total cellular protein extracts. The protein concentrations were determined using Bradford method [24]. Equivalent amounts of proteins (50 *μ*g) were separated by SDS-PAGE and electrotransferred to supported nitrocellulose membranes (Amersham, Sweden) by a semidry transferor. After the membranes were blocked for 2 hours in blocking buffer (5% skimmed milk in PBS-T containing 0.05% Tween 20) at RT, they were incubated with the different primary antibodies: rabbit polyclonal anti-mTOR and p70S6K, mouse monoclonal anti-4EBP1 and p-4EBP1 of 1 : 200 and *β*-actin of 1 : 400 (Santa Cruz Biotechnology, USA), and mouse monoclonal anti-p-p70S6K of 1 : 2,000 (Cell Signaling Technology, USA) diluted in 1% skimmed milk in PBS-T, respectively, at RT for 2 hours, followed by being incubated with the appropriate HRP-linked secondary antibodies. Finally, the bands of specific proteins on the membranes were visualized with chemiluminescent substrate (Santa Cruz Biotechnology, USA) according to the manufacturer's instructions. The membranes were rinsed three times with PBS-T between the incubations described above [18].

### 2.7. Semiquantitative RT-PCR

Total RNA was prepared from the xenograft tissues with trizol reagent and reversely transcribed to cDNA using AMV First Strand DNA Synthesis Kit (Biotech Co., Shanghai, China). Briefly, a 1 *μ*g of the isolated RNA was reversely transcribed to cDNA at 37°C for 1 hour in a 20 *μ*L of reaction mixture containing 1 *μ*L AMV reverse transcriptase, 1 *μ*L random hexamer, 4 *μ*L 5 × AMV buffer, 1 *μ*L RNase inhibitor (20 U/*μ*L), and 2 *μ*L dNTP (10 mM). The PCR amplification mixture of 25 *μ*L contained 0.5 *μ*L cDNA mixture, 0.5 U Taq DNA polymerase, 2.5 *μ*L of 10 × PCR buffer, 2.5 mM dNTP mixture, and 50 pM sense and antisense primers each. The used oligonucleotide primers of mTOR, p70S6K, and 4EBP1 and the PCR conditions were as described previously [18]. The amplified products were subjected to electrophoresis on 1% agarose gels containing 0.2 *μ*g/*μ*L ethidium bromide and visualized under a UV light [18].

### 2.8. TUNEL Assay

Terminal deoxynucleotidyl transferase dUTP nick end labeling (TUNEL) staining was carried out using the* in situ *cell death detection kit (KeyGen Biotech Ltd., China) according to the manufacturer's instructions. Briefly, xenograft tissues were embedded in paraffin and then sectioned for the TUNEL assay. The tissue sections were deparaffinized in xylene and rehydrated in graded ethanol for dehydration. After being washed with PBS and incubated in 3% H_2_O_2_ solution for 20 minutes, the sections were treated with proteinase K (20 *μ*g/mL in PBS) for 20 minutes at RT and rewashed with PBS. Subsequently, the sections were treated with a biotin-dNTP reaction mixture labeled by TdT at 37°C for 1 hour and treated with streptavidin-HRP solution for 5 minutes at RT. Finally, the slides were washed and developed with DAB solution and counterstained with hematoxylin. The results were determined by counting 1,500 cells in 5 randomly selected fields.

### 2.9. Statistical Analysis

The results of all experiments were performed by standard chi-square test and one-way analysis of variance, respectively, using SPSS version 20.0 (SPSS, Chicago, USA). All summary statistics were expressed as means ± SD but tumor volumes were expressed as means ± SE. In all statistical analyses, a *P* value <0.05 was considered statistically significant.

### 2.10. Study Ethics Approval

The study was approved by the Ethics Committee of Zhengzhou University, Henan, China.

## 3. Results

### 3.1. Expression of mTOR in ESCC Tissues

**Figure 1:**
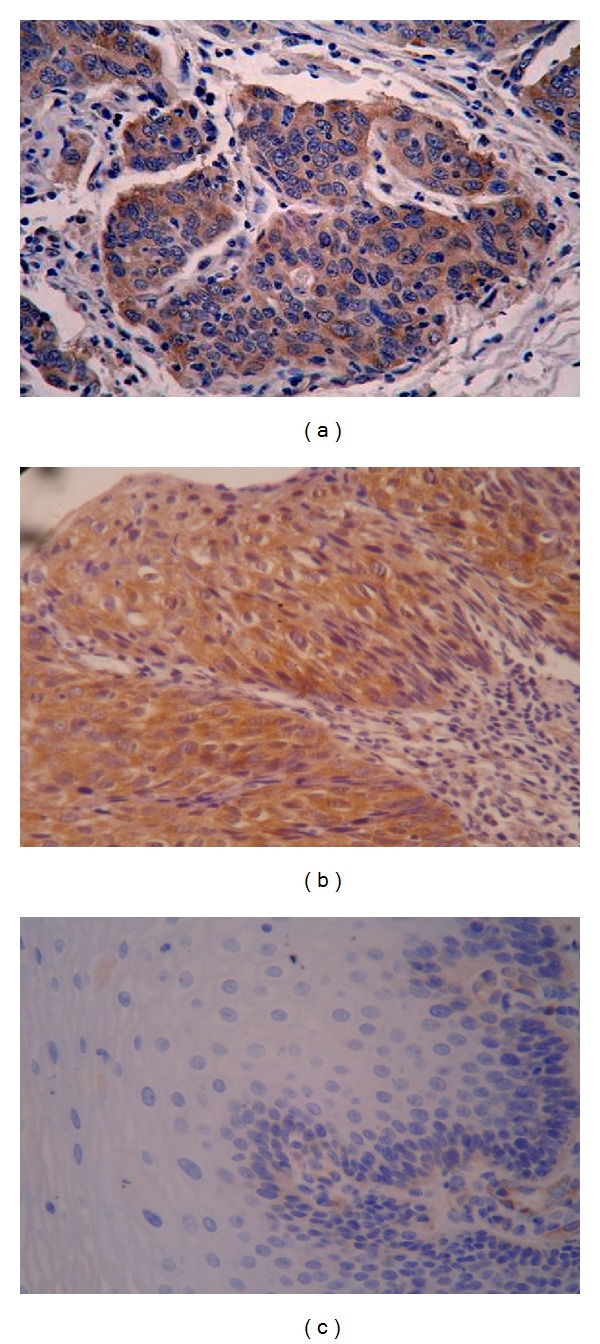
Expression of mTOR in human normal esophageal and ESCC tissues by immunohistochemical analysis. (a) Negative expression of mTOR in normal tissues of the esophagus. (b) Moderate positive expression of mTOR in dysplasia tissues. (c) Positive expression of mTOR in ESCC tissues (×400).

To examine the potential role of the mTOR pathway in ESCC, the expression of mTOR was examined immunohistochemically in ESCC tissues, and the results showed that mTOR was mainly expressed in the cytoplasm (Figure 1). The expression rates of mTOR were 20% (3/15), 46.7% (7/15), and 62.9% (22/35) in normal esophageal, dysplasia, and cancer tissues, respectively. As shown in Tables 1 and 2, there was a statistical significance among them (*P* < 0.05). The expression of mTOR was not related to the histologic type, the depth of infiltration, and lymph node metastasis (all *P* > 0.05) but closely related to the TNM stage (*P* < 0.01).

### 3.2. Effect of mTOR siRNA on Sensitivity of Cell Proliferation to Cisplatin

**Figure 2:**
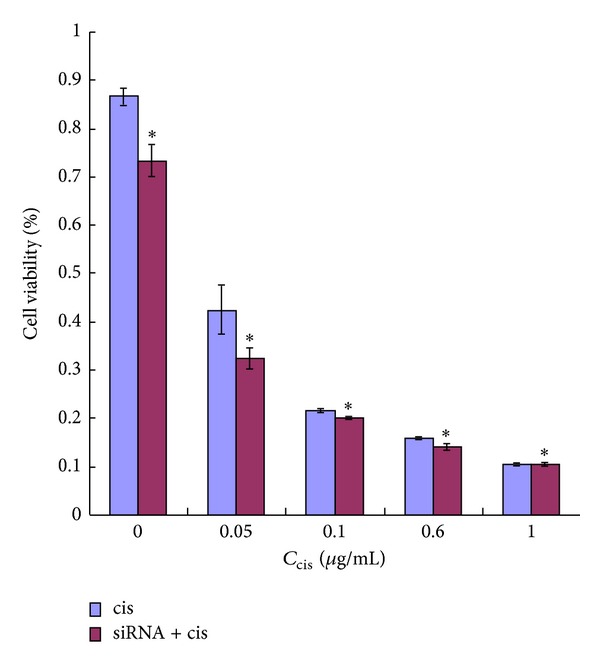
Effects of mTOR siRNA on cell proliferation and sensitivity to cisplatin. To determine the effects of mTOR siRNA on cell proliferation and sensitivity of ESCC cells with mTOR siRNA to cisplatin, proliferations of the cells with or without mTOR siRNA treated with cisplatin at different concentrations for 24 h were detected with WST-8 dye. Results pooled from three independent experiments were expressed as mean ± SD. *P < 0.05, compared to untreated cells.

To investigate the changes of sensitivity of cells to cisplatin after being transfected with mTOR siRNA, the EC9706 cells with or without mTOR siRNA were seeded in a 96-well flat-bottomed plate, cultured at 37°C for 24 hours, and treated with cisplatin of different concentrations of 0.05, 0.1, 0.6, and 1 *μ*g/mL for 24 hours. As shown in Figure 2, the proliferation of cells with or without mTOR siRNA became slower and inhibitory effects of cisplatin on proliferation of EC9706 cells were in a dose-dependent manner. The ratio of alive cells with mTOR siRNA was lower (*P* < 0.05) than that without mTOR siRNA at the same concentration of cisplatin. But the proliferations revealed no difference between cells with and without siRNA in presence of 1 g/mL cisplatin.

### 3.3. Inhibition Effects of Cisplatin and mTOR siRNA on the Growth of Xenografts

**Figure 3:**
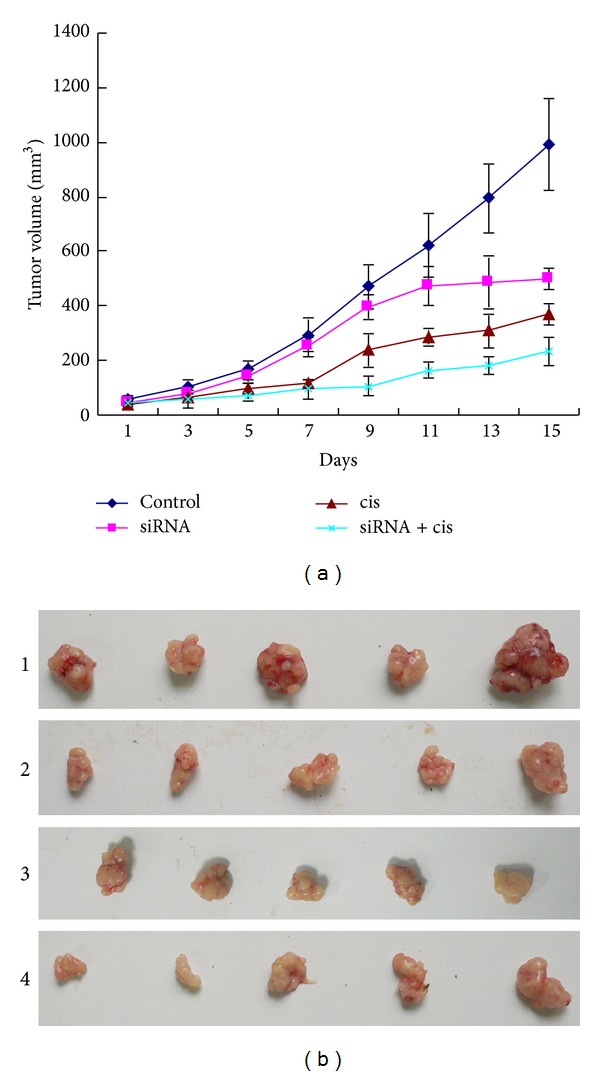
Tumor regression observed in EC9706 xenografts treated with different ways. (a) Tumor volumes from the xenografts of groups each were assessed every day, as described in Materials and Methods, and the results were expressed as means ± SE (mm^3^). The tumor growth of treated groups each became slow, in which group treated with mTOR siRNA combined with cisplatin was the slowest. (b) Tumors from the xenograft treated with different ways for two weeks. 1, 2, 3, and 4 represent control, mTOR siRNA, cisplatin, and mTOR siRNA + cisplatin groups, respectively.

The sensitivity of xenografts with or without mTOR siRNA to cisplatin was evaluated in the transplantable tumor of ESCC. As shown in Table 3 and Figure 3, the volumes of tumors in all groups were progressively increased during the experiment, of them the volume of tumors in the control group on day 15 after the treatment was 17-fold bigger (*P* < 0.01) than that on day 1. Antitumor effect of mTOR siRNA and cisplatin alone or combined with each other emerged from days 7 to 15 at termination of the treatment, which had statistic difference between the three experimental groups and the control group (day 7: *P* < 0.05; days 9–15: all *P* < 0.01). The volume of tumors on day 15 after the treatment was 9-fold bigger than that on day 1 in mTOR siRNA or cisplatin alone group (*P* < 0.05), while the volume of tumors in mTOR siRNA combined with cisplatin group on day 15 after the treatment was 5-fold bigger than that on day 1 (*P* < 0.01). Additionally, compared to control group, the inhibition rate of tumor growth was 58.27% after mTOR siRNA alone treatment and 63.18% after single-agent cisplatin, respectively, while combination of mTOR siRNA with cisplatin significantly enhanced the inhibition effect of tumor growth with 76.70% on ESCC in vivo at termination of the experiment. Obviously, the treated groups each showed significant growth inhibition compared to control group (*P* < 0.05 or < 0.01) during the same period, especially the mTOR siRNA + cisplatin group, indicating that mTOR siRNA combined with cisplatin has the strongest inhibition of tumor growth.

### 3.4. Actions of mTOR siRNA and Cisplatin on Effectors in mTOR Pathway

**Figure 4:**
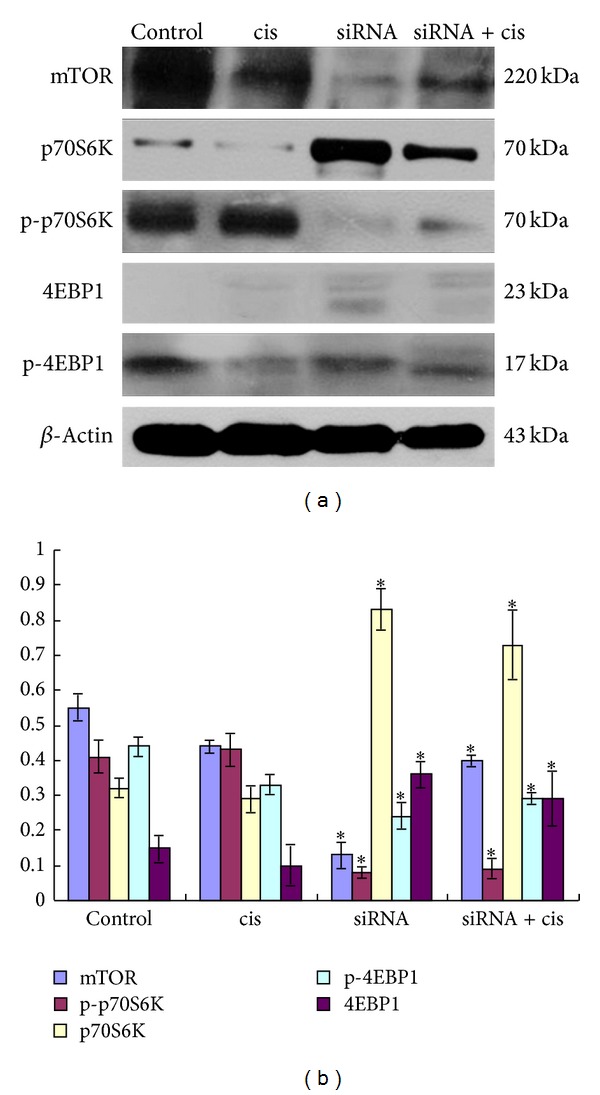
Protein expression of effectors in mTOR pathway of EC9706 xenografts treated with different ways. (a) Antibodies to mTOR, p70S6K, p-p70S6K, 4EBP1, and p-4EBP1, respectively. (b) Semiquantitative values of bands from three independently repeated experiments, which were statistically analyzed by densitometry using TotalLab 2.0 software, are expressed as means ± SD. Reduced expressions of mTOR, p-p70S6K, and p-4EBP1 and elevated expressions of p70S6K and 4EBP1 were observed in groups treated with mTOR siRNA and mTOR siRNA combined with cisplatin. *β*-Actin was used as loading control. *P < 0.05, compared to control group.

mTOR and the phosphorylation status of its downstream targets, p70S6K and 4EBP1, in the xenografts were examined by western blots. As shown in Figure 4, mTOR siRNA alone and mTOR siRNA combined with cisplatin downregulated the protein levels of mTOR, p-p70S6K, and p-4EBP1 but upregulated the protein levels of p70S6K and 4E-BP1 (*P* < 0.05). Compared to the control group, the protein expression levels of mTOR, p-p70S6K, and p-4EBP1 had no obvious changes in cisplatin group (*P* > 0.05), while they decreased by ~4-fold, ~5-fold, and ~2-fold, respectively, in the mTOR siRNA group, and 1.4-fold, 4.5-fold, and 1.5-fold, respectively, in mTOR siRNA + cisplatin group, demonstrating that the protein expression of mTOR between the mTOR siRNA group and mTOR siRNA + cisplatin group has a difference (*P* < 0.05) but not p-p70S6K and p-4EBP1 (*P* > 0.05).

**Figure 5:**
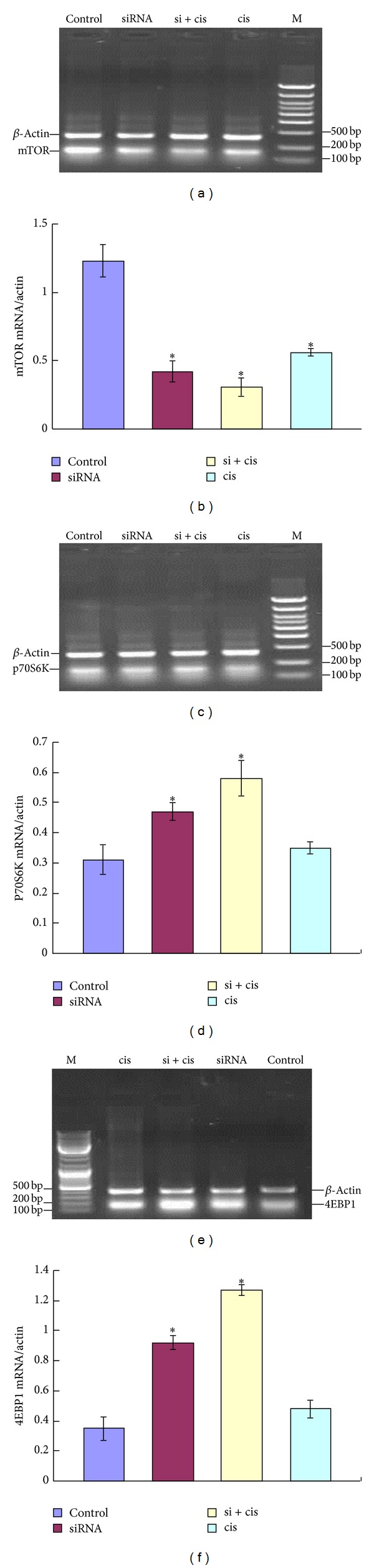
Analysis of total mRNA expressions of mTOR (a), p70S6K (b), and 4EBP1 (c) from EC9706 xenografts treated by different ways. ((d)–(f)) Semiquantitative values of mRNA levels of mTOR, p70S6K, and 4EBP1 to *β*-actin, respectively. Results from three independently repeated experiments, which were statistically analyzed by densitometry using BandScan 5.0 software, are expressed as means ± SD. The mRNA level of mTOR was downregulated while mRNA levels of p70S6K and 4EBP1 were upregulated after being treated with mTOR siRNA and siRNA + cisplatin. *P < 0.05, compared to control group.

To investigate the effect of mTOR siRNA and cisplatin alone or combined with each other on the mRNA expressions of the effectors in mTOR pathway, their mRNA expressions in all groups were measured by RT-PCR. Compared to the control group, the mRNA level of mTOR was downregulated while the levels of p70S6K and 4EBP1 mRNA were upregulated in mTOR siRNA alone or siRNA combined with cisplatin groups (*P* < 0.05). It had no obvious changes in cisplatin alone group (*P* > 0.05, Figure 5), suggesting that cisplatin has no effect on mTOR pathway.

### 3.5. Effects of mTOR siRNA Alone or Combined with Cisplatin on Cell Apoptosis

The apoptosis of tumor cells of ESCC xenografts in groups each was determined by an* in situ* TUNEL assay and the results showed that there were 63, 54, and 102 apoptotic cells/1,500 cells in mTOR siRNA, cisplatin, and mTOR siRNA + cisplatin groups, respectively, compared to control group (6 apoptotic cells/1,500 cells) (*P* < 0.05 or <0.01, Table 4). The number of apoptosis cells in mTOR siRNA + cisplatin group was the highest among the three experimental groups (*P* < 0.05), while the number of apoptosis cells in mTOR siRNA group had no statistical difference from that of cisplatin group (*P* > 0.05). The results above indicate that mTOR siRNA promotes apoptosis of ESCC cells and the effect of inducing apoptosis is stronger when it is combined with cisplatin.

## 4. Discussion

Since mTOR was identified and cloned in 1994 [25], it has been examined in a wide array of cancer types and aberrantly activated mTOR pathway plays an essential role in the growth of different types of tumors including ESCC [26, 27]. So far, several statuses of the effectors on the upstream and downstream of mTOR pathway such as amplification of a catalytic subunit of PI3K and loss or mutation of PTEN gene have been detected in many malignant tumors [28, 29]. In some malignancies, proteins lying downstream of the mTOR pathway have been also altered, for example, eIF4E, which promotes the phosphorylation of 4EBP1 (p-4EBP1), expression levels of which correlate with tumor progression [28].

The activation of mTOR has been shown in ESCC cell lines in our previous study [20]. In this study, we examined the expression of mTOR in ESCC specimens and investigated the relationship of the expression level of mTOR with infiltration, lymph node metastasis, and TNM phase. As shown in Figure 1 and Tables 1 and 2, the expression rate of mTOR of cancer tissue was the highest (62.9%) and there were statistically significant differences among normal esophageal, dysplasia, and cancer tissues (*P* < 0.05). Moreover, the expression of mTOR was closely related to the TNM phase (all *P* < 0.01). The above-mentioned results indicate that the aberrantly activated mTOR may be a clinical diagnostic mark in ESCC.

As the special inhibitors of mTOR, rapamycin and its anologs have been evaluated in many tumors including ESCC and have shown the marked inhibition effects on the mTOR pathway and tumor growth [12, 13, 17]. RNA silencing including both short interfering RNA (siRNA) and short hairpin RNA (shRNA) has been used in cancer research in vitro and in vivo [30–32]. In this study, we found that mTOR siRNA alone could lead to slow growth of tumors and the volume of tumors at termination of the experiment on day 15 was only 9-fold bigger than that on day 1 in the group of mTOR siRNA alone, but 17-fold in the control group, and there was a significant difference (*P* < 0.05) in comparison of mTOR siRNA alone with control groups. As demonstrated by TUNEL assay, moreover, the number of apoptotic cells in mTOR siRNA group was 10-fold more than that in control group, suggesting that mTOR siRNA alone may induce cell apoptosis of ESCC.

Cisplatin is a widely used chemotherapeutic agent that exerts its cytotoxic effects by disrupting the DNA structure in cells through the formation of intrastrand adducts and interstrand cross-links [33]. It has been proven to be one of the most clinically active agents for the treatment of a variety of solid tumors, including ESCC [34]. However, its clinical therapeutic effect is often limited by intrinsic or acquired tumor cell resistance. In addition, cisplatin's associated nephrotoxicity and neurotoxicity, especially when administered at higher doses, have been further obstacles to the success of this treatment [30]. Thus, we investigated the combination effect of mTOR siRNA and cisplatin to determine whether mTOR siRNA would potentiate the effects of cisplatin on ESCC. The results of cells proliferation showed that cisplatin that inhibited the proliferation of EC9706 cells was in a dose-dependent manner and the inhibition effects of cisplatin became stronger at the same concentration after cells were transfected with mTOR siRNA (*P* < 0.05). But the proliferations of cells with/without siRNA revealed no difference in presence of 1 g/mL cisplatin, which may be because the inhibition ratio of cisplatin at this concentration was very high, nearly reached 90% and made the role of mTOR siRNA seem to be not obvious. The results of nude mice showed that the tumors growth became slow when cells were treated with mTOR siRNA and cisplatin alone or combined compared to control group from day 7 to 15 after treatment, which had statistic differences between the three experimental groups and the control group; while the tumor growth was the slowest, when mTOR siRNA combined with cisplatin, the volume of tumor only was 5-fold bigger in the mTOR siRNA combined with cisplatin group but 17-fold bigger in the control group at the termination than at day 1 of the treatment (*P* < 0.01). The inhibition rates of tumors in mTOR siRNA, cisplatin, and mTOR siRNA + cisplatin groups were 58.27%, 63.18%, and 76.70%, respectively, indicating that mTOR siRNA combined with cisplatin has the strongest inhibition of tumor growth. Additionally, the number of apoptosis cells was the most in mTOR siRNA + cisplatin group (102 cells/1,500 cells) compared to that in mTOR siRNA (63 cells/1,500 cells) and cisplatin (54 cells/1,500 cells) alone group (*P* < 0.05). A potential explanation for the result is that mTOR siRNA inhibits the phosphorylation of p70S6K and 4EBP1, the key factors in cell proliferation and growth, so cisplatin-induced cell proliferation and apoptosis are enhanced. Above all, mTOR pathway has important effects on the tumorigenesis and progression of ESCC and inhibition of mTOR pathway by mTOR siRNA promotes the sensitivity of cells to cisplatin.

In this study, mTOR was overexpressed in ESCC specimens compared to normal esophageal tissue and the expression level of mTOR had close relationship to the TNM phase of ESCC. Transient inhibition of mTOR by mTOR siRNA significantly increased the sensitivity of the EC9706 cells to cisplatin in vitro and in vivo. Besides, cisplatin significantly inhibited in vivo growth of ESCC xenografts and increased the number of apoptotic cell after mTOR pathway was inhibited by mTOR siRNA. The above results indicate that mTOR may be a potential therapeutic target and inhibition of mTOR pathway can improve the sensitivity of chemotherapeutics in ESCC.

## Figures and Tables

**Table 1:** Expressions of mTOR proteins in different tissues.

Tissue type	*n*	mTOR			*P*
		−	+	Positive rate (%)	
Normal	15	12	3	20.0	0.021
Dysplasia	15	8	7	46.7	
Cancer	35	13	22	62.9	

**Table 2:** Clinical significance of mTOR protein expression.

Pathological features	*n*	mTOR	
		Positive *n* (%)	*P*
Histology classification			
I	9	6 (66.7)	0.917
II	14	9 (64.3)	
III	12	7 (58.3)	
Depth of infiltration			
Mucosa	7	3 (42.9)	0.308
Muscle layer	15	9 (60.0)	
Fiber membrane	13	10 (76.9)	
Lymph node metastasis			
No	19	10 (52.6)	0.172
Yes	16	12 (75.0)	
TNM phase			
I, II	13	4 (30.8)	0.003
III, IV	22	18 (81.8)	

**Table 3:** Effects of mTOR siRNA and cisplatin alone or combined on growth of human ESCC xenograft in nude mice (*n* = 5).

Group	Animal weight	Tumor volume at beginning^1^	Tumor volume at termination	Inhibition rate of tumor (%)
Control	20.54 ± 1.23	57.03 ± 10.45	999.28 ± 167.30	—
siRNA	20.52 ± 2.08	43.62 ± 10.85	416.97 ± 38.12*	58.27
cis	20.38 ± 1.42	39.09 ± 2.08	367.91 ± 61.21*	63.18
siRNA + cis	20.66 ± 1.79	46.28 ± 25.56	232.86 ± 54.23*	76.70

^1^Tumor volume is expressed as mm^3^. *P < 0.05, compared to control group.

**Table 4:** Effects of mTOR siRNA alone or combined with cisplatin on cell apoptosis (TUNEL).

Group	The counted cell number	The number of apoptosis cells
Control	1500	6 ± 1
siRNA	1500	63 ± 2*
cis	1500	54 ± 5*
siRNA + cis	1500	102 ± 4*

*P < 0.05, compared to control group.
